# CNS Manifestations in Mucolipidosis Type II—A Retrospective Analysis of Longitudinal Data on Neurocognitive Development and Neuroimaging in Eleven Patients

**DOI:** 10.3390/jcm12124114

**Published:** 2023-06-18

**Authors:** Luise Sophie Ammer, Karolin Täuber, Anna Perez, Thorsten Dohrmann, Jonas Denecke, René Santer, Ulrike Blümlein, Ann-Kathrin Ozga, Sandra Pohl, Nicole Maria Muschol

**Affiliations:** 1 International Centre for Lysosomal Disorders (ICLD), Department of Paediatrics, University Medical Centre Hamburg-Eppendorf, 20246 Hamburg, Germany; k.taeuber@uke.de (K.T.); a.perez@uke.de (A.P.); j.denecke@uke.de (J.D.); r.santer@uke.de (R.S.); s.pohl@uke.de (S.P.); muschol@uke.de (N.M.M.); 2 Department of Paediatric Intensive Care and Neonatology, Altona Children’s Hospital, 22763 Hamburg, Germany; 3 Department of Child and Adolescent Psychiatry and Psychotherapy, University Medical Center Hamburg-Eppendorf, 20246 Hamburg, Germany; 4 Department of Anaesthesiology, University Medical Centre Hamburg-Eppendorf, 20246 Hamburg, Germany; t.dohrmann@uke.de; 5 Department of Paediatrics, Carl-Thiem-Klinikum gGmbH, 03048 Cottbus, Germany; u.bluemlein@ctk.de; 6 Institute of Medical Biometry and Epidemiology, University Medical Centre Hamburg-Eppendorf, 20246 Hamburg, Germany; a.ozga@uke.de; 7 Department of Osteology and Biomechanics, University Medical Centre Hamburg-Eppendorf, 20246 Hamburg, Germany

**Keywords:** mucolipidosis, ML, MLII, natural history, neurocognition, adaptive behaviour, development, imaging, MRI, CNS

## Abstract

Mucolipidosis type II (MLII), an ultra-rare lysosomal storage disorder, manifests as a fatal multi-systemic disease. Mental inhibition and progressive neurodegeneration are commonly reported disease manifestations. Nevertheless, longitudinal data on neurocognitive testing and neuroimaging lack in current literature. This study aimed to provide details on central nervous system manifestations in MLII. All MLII patients with at least one standardized developmental assessment performed between 2005 and 2022 were included by retrospective chart review. A multiple mixed linear regression model was applied. Eleven patients with a median age of 34.0 months (range 1.6–159.6) underwent 32 neurocognitive and 28 adaptive behaviour assessments as well as 14 brain magnetic resonance imagings. The scales used were mainly BSID-III (42%) and VABS-II (47%). Neurocognitive testing (per patient: mean 2.9, standard deviation (SD) 2.0) performed over 0–52.1 months (median 12.1) revealed profound impairment with a mean developmental quotient of 36.7% (SD 20.4) at last assessment. The patients showed sustained development; on average, they gained 0.28 age-equivalent score points per month (confidence interval 0.17–0.38). Apart from common (63%) cervical spinal stenosis, neuroimaging revealed unspecific, non-progressive abnormalities (i.e., mild brain atrophy, white matter lesions). In summary, MLII is associated with profound developmental impairment, but not with neurodegeneration and neurocognitive decline.

## 1. Introduction

Mucolipidosis type II (MLII, OMIM #252500), an ultra-rare lysosomal storage disorder, is caused by mutations in the *GNPTAB* gene, which encodes the alpha/beta-subunit precursor of the N-acetylglucosamine (GlcNAc)-1-phosphotransferase [1]. The GlcNAc-1-phosphotransferase is located in the Golgi apparatus and responsible for the first step of the sequential formation of mannose 6-phosphate (M6P) residues on newly synthesized lysosomal enzymes [2]. M6P residues are required for intracellular targeting of these enzymes to the lysosomes. In cells from patients with MLII, the activity of GlcNAc-1-phosphotransferase is significantly reduced, which causes mis-sorting of multiple lysosomal enzymes, hypersecretion into the extracellular space and their subsequent intralysosomal deficiency. Consecutively, non-degraded macromolecules (i.a., glycosaminoglycans, lipids, cholesterol) accumulate within lysosomes, compromising cellular function and leading to tissue damage [3].

Clinically, MLII manifests as a progressive multi-systemic disease with prenatal or neonatal onset. Major clinical features are craniofacial dysmorphia with gingival hyperplasia, cardiac and respiratory dysfunction, hepatosplenomegaly, and distinct skeletal pathologies. The latter comprise severe growth inhibition, skull, spine, thoracic and long tubular bone deformities, hip dysplasia, clubfeet, and joint contractures [3,4,5,6,7]. In addition to the somatic disease burden, MLII patients present with mental disabilities [3,4,8] and an intelligence quotient of below 85 [9]. MLII is regularly listed among lysosomal storage disorders associated with neurodegeneration and cognitive decline [3,8,10,11,12,13]. A recently published systematic review confirmed an early fatal outcome with a median age of death of 1.8 years [4], usually due to cardiopulmonary complications. To date, no causal treatment exists for MLII, not even in the framework of clinical trials. Experimental haematopoietic stem cell transplantation (HSCT) is a therapeutic approach for rare neurodegenerative lysosomal storage disorders [14,15,16] and was also performed in few patients with MLII [17,18]. A surge of innovative treatments (i.e., anti-inflammatory drugs, gene therapy, antisense oligonucleotides) were tested in cell culture and animal models [19,20,21]. 

Progressive mental inhibition and neurodegeneration are commonly reported disease manifestations in MLII, but longitudinal data based on formal neurocognitive testing and neuroimaging are missing in current literature. Better knowledge on the natural history of MLII might improve clinical patient management including supportive therapies. Moreover, considering that MLII is yet untreatable, detailed information on central nervous system (CNS) manifestations of therapy-naive patients is indispensable as reference data to be able to assess effects of experimental or upcoming therapies. This study aimed to provide comprehensive information on neurocognitive development and adaptive behaviour as well as on neuroimaging features in MLII by systematic analysis of standardized developmental assessments and magnetic resonance imaging (MRIs) of the brain and cervical spine. 

## 2. Methods

### 2.1. Study Site and Patients

This study was conducted by a retrospective chart review of patients of the International Centre for Lysosomal Disorders (ICLD) located at the University Medical Centre Hamburg-Eppendorf (UKE), Hamburg, Germany. Inclusion criteria were clinically and biochemically or molecular genetically confirmed diagnosis of MLII and at least one standardized developmental assessment performed between September 2005 and May 2022. Patients who presented a less severe phenotype than expected in classical MLII were categorized as ML intermediate. As patients with ML intermediate still manifested severe multi-systemic symptoms [9], they were included in the MLII patient cohort. Most of the patients presented here were already described concerning their somatic disease burden, with foci on hip pathologies and anaesthesia-relevant disease manifestations [5,7].

### 2.2. Measures

Data acquisition was performed by systematic review of analogue and electronic patient health records. From each patient, we collected baseline characteristics including sex, genotype, clinical phenotype, body size metrics, and age at diagnosis and death (if applicable). As part of the standard of care of the ICLD, the patients underwent regular multidisciplinary diagnostic workups including developmental assessments, audiograms, and ophthalmological examinations. 

All well-proven standardized series of developmental assessments [22] performed during the study period were included, namely Bayley Scales of Infant and Toddler Development-Second/Third Edition (BSID-II/-III), Griffith Scales of Child Development-Third Edition (GMDS-III), Snijders–Oomen Nonverbal Intelligence Test—Revised for children up to seven years of age (SON 2.5–7), and Kaufman Assessment Battery for Children—Second Edition (KABC-II). Denver Developmental Screening Tests were excluded for the matter of precision. The Vineland Adaptive Behaviour Scales—Second Edition (VABS-II), performed by parent/caregiver interviews, were enclosed to provide information on adaptive function. The assessment tools typically produce raw scores (i.e., points earned based on the number of items achieved), age-equivalent scores (Aeqs; score that healthy children usually achieve at the respective age), and developmental quotients (DQs; Aeqs divided by the chronological age times 100). The BSID-III methodically covers Aeqs of up to 42 months [22]. For analysis of the BSID-III, the German standardization of the scale was used. For the calculation of cognitive Aeqs based on the VABS-II, the motor skill domain was neglected to prevent falsification by physical restrictions. Hence, according to our previous work [17], a mean of the three remaining domains (activity of daily living, communication, social interaction) was drawn for this purpose. 

The patients’ developmental test results and records were, furthermore, systematically screened for data on the following aspects: musculoskeletal burden; ability to see, hear, speak, sit and walk; placement of a tracheostoma or gastric/nasal tube; history of seizures or psychiatric symptoms. The degree of hearing impairment was defined as per audiology as follows: minor, hearing threshold at 25–40 decibel (dB); moderate, threshold at 40–60 dB; severe, threshold at 60–80 dB. 

We collected all MRIs of the neurocranium and cervical spine performed in the patients during the study period. The original images were reviewed by an experienced neuropaediatrician, who filled out a standardized evaluation form.

### 2.3. Statistics

Data were collected in Microsoft Excel (Version 2011, Microsoft Corporation, Redmont, WA, USA) and analysed in R 4.1.2 (R core team, Vienna, Austria). Categorical variables are summarized as frequencies and percentages, continuous variables as medians with minimum and maximum and means with standard deviations (SD). A multiple mixed-effects linear regression model was applied to account for the repeated measurements in each patient (i.e., random intercept) using the Ime4 package in R [23]. Thereby, the Kenward–Roger method was used. The analysis of the developmental trend was exclusively based on “objective” developmental assessments (BSID-II/-III; GMDS-III; SON 2.5–7; KABC-II); hence, “subjective” parent-based assessments (VABS-II) were excluded from the model. We investigated the interaction between the chronological age and the phenotype, in order to be able to distinguish between the two phenotypes MLII and ML intermediate. This interaction was excluded in case of an interaction *p*-value of >0.05. Marginal means with corresponding 95%-confidence intervals (CI) were calculated, as well as the intra-class correlation (ICC). Model assumptions were checked graphically (i.e., a histogram of residuals and qq-plot). The Spearman correlation (r) between BSID-III and VABS-II values was calculated. As this was an explorative study, neither model validation nor adjustment for multiple testing or imputation of missing values was performed.

## 3. Results

### 3.1. Data Acquisition

**Figure 1:**
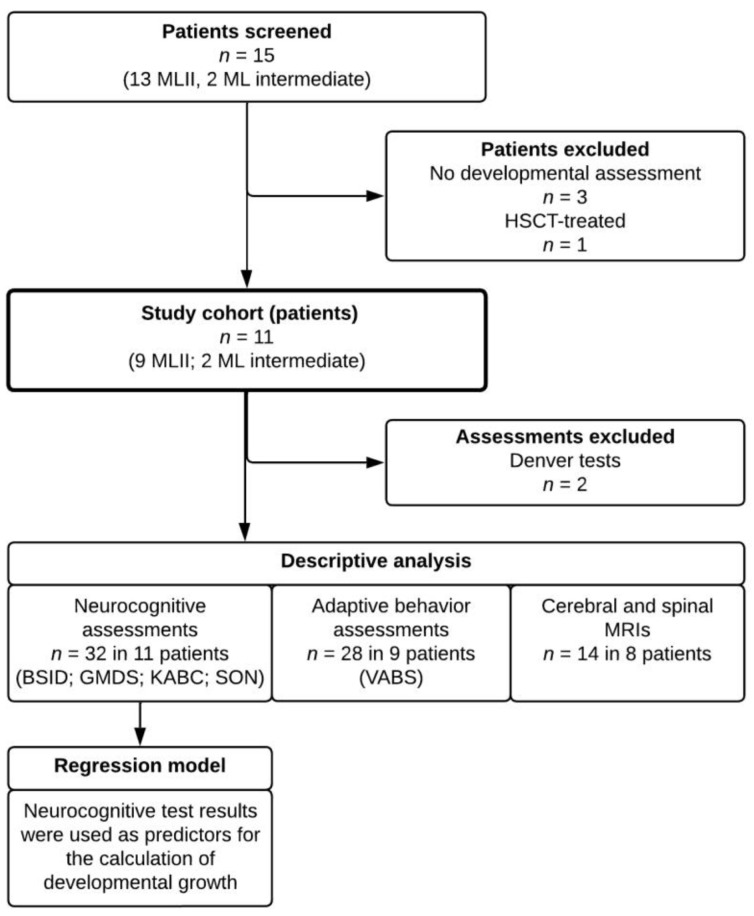
Flowchart of the study inclusion process. Abbreviations: BSID, Bayley Scales of Infant and Toddler Development-II/III; GMDS; Griffith Scales of Child Development-III; HSCT, haematopoietic stem cell transplantation; KABC, Kaufman Assessment Battery for Children-II; ML, mucolipidosis; MRIs, magnetic resonance imaging examinations; SON, Snijders-Oomen Nonverbal Intelligence Test-2.5-7; VABS, Vineland Adaptive Behaviour Scales-II.

Eleven patients (nine MLII, two ML intermediate) with at least one standardized developmental assessment available were enrolled in the study accounting for a total of 60 developmental assessments and 14 MRI examinations. The study inclusion process is summarized in Figure 1.

### 3.2. Patient Characteristics

#### 3.2.1. Patient Baseline Characteristics

The predominant phenotype in our study population was MLII in nine patients (82%). Two patients (18%) presented a slightly attenuated disease course and were, hence, phenotypically categorized as ML intermediate (Table 1). Sex distribution was balanced with six males (55%) and five females (45%). The median overall age was 29.1 months (range 1.6–66.5) in MLII patients and 148.1 months (range, 75.0–159.6) in ML intermediate patients. The diagnosis was confirmed at a median age of 14.6 months (range 0.8–27.7). By the time of analysis, four patients (36%) died at an age between 3.1 and 11.8 years. The *GNPTAB* genotype was known in nine patients (82%) and included missense (c.10A > C, c.1001G > A), nonsense (c.3091C > T), frameshift (c.344_345del, c.3503_3504del, c.1052dup, c.2502del, c.1022del), and splice site (c.3335 + 1G > A) variants, which were previously described in other patients with MLII [3].

#### 3.2.2. Somatic Disease Burden

The somatic burden was overall high. All study patients suffered from severe orthopaedic problems at all ages, specifically dysostosis multiplex with spine and long bone deformities, severe joint contractures, and profound growth inhibition with a mean height of 70.0 cm (SD 10.0) at last observation. Hip dysplasia was documented in nine patients (81%) and carpal tunnel syndrome in two (18%). Affections of the middle ear were evident in all but one of the children (91%). Eight of those children underwent ear, nose, and throat surgery. Sensoneuronal hearing impairment was confirmed in six patients (55%; minor *n* = 2, severe *n* = 4) and suspected but unproven in three further patients. Seven patients manifested at least one ophthalmological problem (63%; refraction disorder/strabismus *n* = 5, mild corneal clouding *n* = 2, retinopathy *n* = 3). 

### 3.3. Neurocognitive and Adaptive Function

#### 3.3.1. Developmental Assessments

Collectively, the study patients underwent 32 neurocognitive and 28 adaptive behaviour assessments. The median age at first developmental assessment was 18.5 months (range 1.6–135.5) and 37.6 months (range 5.6–159.6) at the last (Table 2). Per patient, a mean of 5.5 developmental assessments (SD 3.83) were carried out over a follow-up period of up to 52.1 months (median 20.9). Of note, neurocognitive and adaptive behaviour testing may have been performed on the same day counting as two separate developmental assessments. Looking at the neurocognitive assessments alone, a mean of 2.9 (SD 2.0) assessments were performed per patient. The neurocognitive test battery was mainly comprised of BSID-III (*n* = 25; 78%), followed by GMDS-III (*n* = 2; 6%), KABC-II (*n* = 2; 6%), SON 2.5–7 (*n* = 2; 6%), and BSID-II (*n* = 1; 3%). All adaptive behaviour assessments were performed by VABS-II. A detailed list of all developmental test results can be retrieved from the Supplementary Materials (Table S1). 

#### 3.3.2. Longitudinal Data on Neurocognitive Development

**Figure 2:**
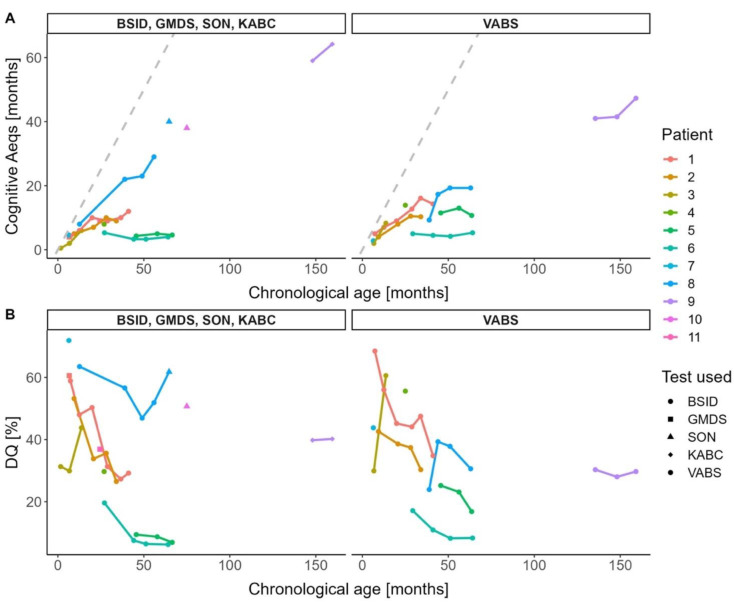
Neurocognitive development in children with MLII and ML intermediate. Developmental curves are visualized as (**A**) cognitive age-equivalent scores (Aeqs) and (**B**) developmental quotients (DQs) in relation to chronological ages. The grey dashed line denotes the development of healthy children. Abbreviations: BSID, Bayley Scales of Infant and Toddler Development-II/III; GMDS, Griffith Scales of Child Development-III; KABC, Kaufman Assessment Battery for Children-II; SON, Snijders-Oomen Nonverbal Intelligence Test-2.5-7; VABS, Vineland Adaptive Behaviour Scales-II.

Neurocognitive testing revealed severe neurocognitive impairment with a mean cognitive Aeqs of 18.1 months (SD 20.0, range 4.0–64.2) and a DQ of 36.7% (SD 20.4, range 6.2–71.9) at last assessment at 6.4–159.6 months of age (median 41.1) (Figure 2). The highest functioning child was a child with ML intermediate, who achieved a cognitive Aeqs of 64.2 months at 159.6 months of age (DQ 40.2%; patient 9). The best-performing child with MLII reached a cognitive Aeqs of 40.0 months at 64.7 months of age (DQ 61.8%; patient 8). Notably, no skills or developmental milestones were lost by any of the children in this study. Children with MLII and ML intermediate both gained skills continuously, on average 0.28 cognitive Aeqs points per month (CI 0.17–0.38) and, therefore, 3.6 times slower than healthy children. The p-value for the interaction between the age and the phenotype was 0.77. Children with intermediate phenotypes scored in mean 18.12 cognitive Aeqs points higher than those with MLII (CI 3.05–33.18). The interclass correlation (ICC) was 0.76; thus, 76% of the total variance was explained by the cluster (i.e., the patients). Overall, DQs declined in all children as a result of an increasing gap with healthy children of the same age. The cognitive test results by BSID-III, which is based on interactions with the child, showed strong correlation (r = 0.76) with the results of the parent-reported VABS-II (Figure S1).

#### 3.3.3. Motor and Verbal Abilities

**Figure 3:**
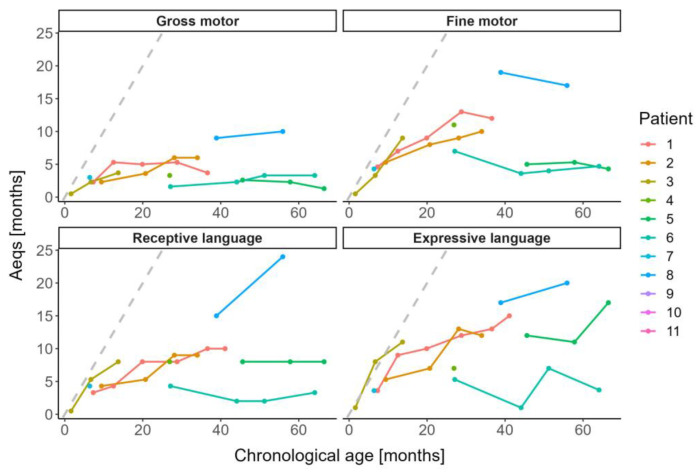
Motor function and verbal abilities in children with MLII and ML intermediate as per Bayley Scales of Infant and Toddler Development-III. The grey dashed line denotes the development of healthy children.

Motor function and communication abilities tended to improve over time; nonetheless gross motor function remained on an extremely low level (Figure 3). The age at last BSID-III assessment ranged between 6.4 and 66.5 months (median 37.6). The mean Aeqs for gross and fine motor function were 4.4 months (SD 2.8) and 8.6 months (SD 4.7) at last BSID-III assessment, respectively. Among the entire cohort, only four patients could sit unsupported (36%; patients 1, 8, 9, 10) and only one patient managed to walk independently (9%; patient 10). The mean Aeqs for receptive and expressive communication abilities were 9.3 (SD 6.4) and 11.2 months (SD 6.1) at last assessment, respectively. Of all patients, the two highest performing children (18%; patients: 9, 10) managed to speak complex sentences. One child (9%; patient 8) could speak 50–80 words and five children (45%; patients 1, 2, 4, 5, 6) 5–10 words, however poorly pronounced. The remaining three (patients 3, 7, 11) did not talk at all, which was still age-appropriate for two of them.

#### 3.3.4. Adaptive Behaviour Abilities

**Figure 4:**
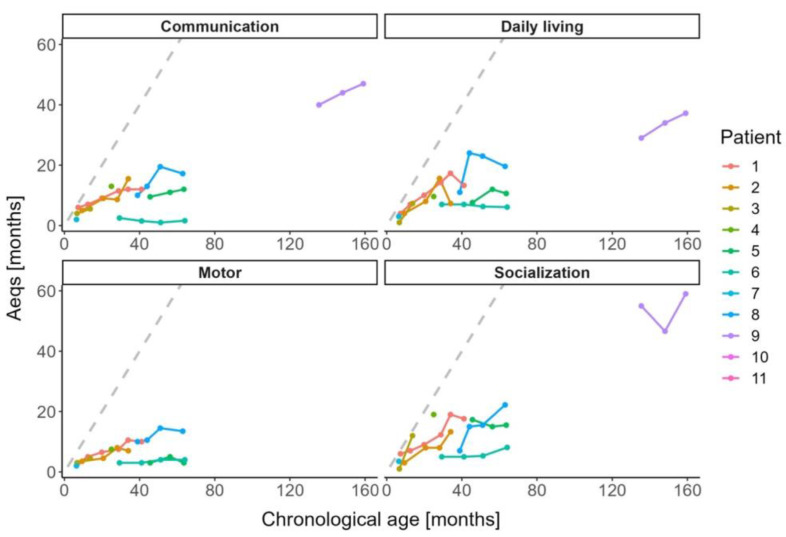
Adaptive behaviour abilities in children with MLII and ML intermediate as per Vineland Adaptive Behaviour Scales-II. The grey dashed line denotes the development of healthy children.

The VABS-II confirmed relative strengths in verbal and social abilities (Figure 4). At a chronological age of 159.6 months, the highest functioning child (ML intermediate; patient 9) captured with the VABS-II managed to socially interact like a healthy 59-month-old child and communicated on a level of a 47-month-old child. However, overall, adaptive behaviour skills, especially those concerning motor function and activities of daily living, remained fairly poor. On the lower end of the phenotypic spectrum, four patients had a history of nasal tube feeding (4/11; 36%; patients 2, 4, 5, 6) and two patients depended on a gastric tube (2/11, 18%; patients 5, 6). None of the patients required tracheostomy. Seizures were not observed in any of the patients. The patients also lacked maladaptive behaviour and obvious psychiatric symptoms other than mental inhibition.

### 3.4. Neuroimaging Features

**Figure 5:**
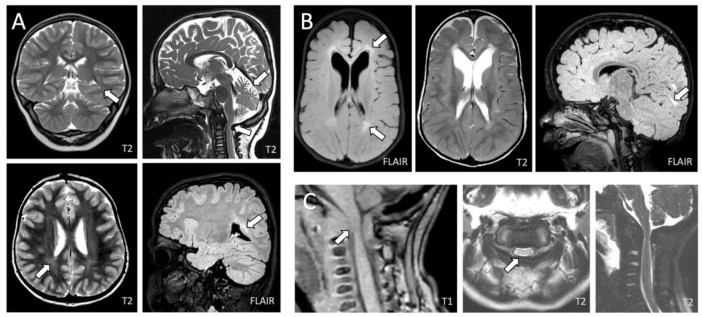
Brain and cervical spine abnormalities found in patients with MLII and ML intermediate via magnet resonance imaging (T2-/T1-weighted and FLAIR sequences). (**A**) Atrophy of the top part of the cerebellum and vermis, cervical spinal canal stenosis, and white matter changes in an 11.2-year-old ML intermediate female (patient 9). (**B**) White matter signal changes in a female 2.4-year-old (patient 6; pictures in transverse plane) and a male 3.2-year-old MLII patient (patient 5; sagittal plane). (**C**) Cervical spinal canal stenosis in a 3.3-year-old MLII male (patient 8).

MRI of the head and cervical spine was performed in eight patients (seven MLII, one ML intermediate; four female, four male). Three children had serial MRI studies with follow-up periods of up to 117.0 months, adding up to an overall of 14 MRIs (per patient: mean 1.8, SD 1.16; range 1–4). The median age at first MRI was 12.6 months (range 7.3–40.7) and 24.0 months (range 7.3–134.7) at the last. All patients examined via MRI presented at least one abnormality of the neurocranium (Table 3). The most common manifestation was the cervical spinal canal stenosis (5/8; 63%), which was progressive in two of the three patients with follow-up MRIs. One ML intermediate patient underwent cervical spine decompression surgery at the age of 2.8 years (patient 9). Mild brain atrophy was seen in four patients (4/8; 50%); however, in one ML intermediate patient, the atrophy was limited to the cerebellum (Figure 5). Brain atrophy lacked clear progression over time in the patients with follow-up examinations. In one MLII patient, the atrophy was milder over time. Other common manifestations were non-progressive atrophy of the white matter (4/8; 50%) and rather progressive T2-hyperintensitive signal abnormalities of the white matter (3/8; 38%), most characteristically found in parieto- and fronto-occipital regions. An impairment of cerebrospinal fluid (CSF) circulation or a hydrocephalus were not observed in any of the children.

## 4. Discussion

MLII is commonly attributed to the two thirds of over 50 lysosomal storage disorders that cause neurodegeneration, cognitive decline, and consecutive death [3,8,10,11,12,13]. However, as an ultra-rare disease, the natural history of MLII is sparsely documented and CNS manifestations in patients with MLII were rarely specified [9,17,24]. To the best of our knowledge, this is the first study to provide objective longitudinal data on neurocognitive function, adaptive behaviour, and neuroimaging abnormalities in a relatively large natural history cohort of children with MLII. The present study features eleven patients with MLII and ML intermediate, who underwent developmental testing and MRI assessments between 1.6 and 159.6 months of age and were followed-up for a maximum of 52.1 months. 

The patients of this study presented with severe neurocognitive impairment with a mean cognitive Aeqs of 18.1 months and a mean DQ of 36.7% at the last assessment. Nevertheless, neurocognitive growth was continuous, in mean 0.28 Aeqs per month. Hence, a neurocognitive decline similar to other lysosomal storage disorders, such as in the mucopolysaccharidoses types IH and IIIA [25,26], may not occur in MLII and/or ML intermediate. Furthermore, it seems the two different phenotypes cannot be distinguished based on their rate of cognitive development considering the insignificant p-value for the interaction between the age and the phenotype (0.77). However, on average, patients with ML intermediate scored 18.12 Aeqs points higher than those with classical MLII. This phenotypical differentiation can only function as a rough estimate considering the large confidence intervals due to the high variability within the phenotypes. The true neurocognitive range might include even more severely impaired children, taking into account such patients were unlikely to have been included, particularly in case of longer transport ways or early death. On the other hand, more than half of the patients were still alive at the time of retrospective analysis. Thus, the actual long-term performance might even be stronger. This might explain why the cognitive disability of MLII patients was categorized as moderate in previous studies [9] and ML intermediate patients were even documented to have normal intelligence [24,27]. 

The patients in this study showed reasonable communication and social skills, but particularly poor motor function. Three patients (27%) were capable to express complex sentences or speak at least 50–80 words. This is in line with a case series by Leroy et al. [24], which documented an interactive and actively playful behaviour and nearly normal language development in patients with MLII. None of the patients in this study surpassed motor function abilities (scores) healthy infants usually achieve. Of all patients, only four could sit unsupported and only a single patient was ambulant. The entire cohort suffered from striking orthopaedic problems (i.a., growth inhibition, bone deformities, hip dysplasia, joint contractures). Afterall, the profound motor function inhibition might rather be attributed to severe orthopaedic manifestations than to impaired cognitive motor planning and executive function. 

Furthermore, almost all study patients had affections of the primary senses, namely auditory (91%) and visual deficits (63%). Children with MLII are known to suffer from recurrent bouts of otitis media and constant nasal discharge [7,28]. This study not only clarified middle ear, but also sensoneuronal hearing loss, which manifested in at least 55% of the patients in this study. Mild corneal clouding was observed in only two patients in this study, which is consistent with the literature; corneal clouding seems to be a late clinical symptom and remains rather faint in MLII [28,29]. However, almost half of the patients actually had visual deficits due to refraction disorders or strabismus. Of note, sleep apnoea was eventually present in all MLII patients [27] and may aggravate a pre-existing developmental delay [30]. As recently published [7], sleep apnoea also prevailed in our patient cohort. None-invasive ventilation improves both neurocognitive outcome and quality of life, even with suboptimal adherence [31]. Conclusively, the high cumulative somatic burden might have a major impact on mental and test performance capacities. This emphasizes the importance of supportive physical and speech therapies and provision of adequate technical aids (i.e., orthoses, hearing devices, none-invasive ventilation) for this patient group. 

Besides common and progressive cervical spinal canal stenosis, no specific brain abnormalities were found in the patients in this study. The high burden of cervical canal stenosis justifies neuroimaging monitoring of patients with ML, in order to allow for risk-benefit considerations of an intervention taking into account the high perioperative morbidity [7,32]. Brain atrophy was present in half of the patients in this study, however mild and non-progressive. In one patient with ML intermediate, brain atrophy was limited to the cerebellum. The clinical relevance of cerebellar atrophy observed in this study remains unclear as the same patient turned out to be the highest functioning child. Notably, we did not perform any volumetry of brain regions, so these results should be generalized with caution. We additionally found common white matter changes. Again, the direct effect of white matter lesions on neurocognitive function remains unclear [33]. The literature contains case descriptions of MLII patients whose hydrocephalus was relieved by the insertion of a ventriculoperitoneal shunt [32,34]. Per contra, none of the patients in this study manifested a hydrocephalus. Alegra et al. [35] also did not detect any hydrocephalus among 15 MLII patients. A hydrocephalus might not be a common complication in ML. The MRI findings of this study were coherent with brain data from animal models (mice, cats) and post-mortem examinations of the brains from humans with MLII, which revealed either no or only mild macroscopic abnormalities [12,36,37]. The case number of this study was too low to deduce a sufficient neuroimaging biomarker for disease severity.

This study had few limitations. The low number of patients was based on limited availability of patients with this ultra-rare disease. Due to the explorative design, the results of the regression model should only be generalized with caution. A limitation of the retrospective study design was the use of different developmental scales, which should only be compared bearing in mind the different test methods. Results of young patients should be interpreted taking into account the low discrimination power of developmental tests at very young ages. Finally, we did not include data on the home environment of the patients that might have affected the neurocognitive outcome. 

## 5. Conclusions

Based on standardized developmental assessments, the present study suggests severe global developmental impairment with particularly low motor function in patients with MLII and ML intermediate. Nevertheless, the longitudinal data indicate sustained neurocognitive growth, albeit very slow. Neuroimaging revealed unspecific abnormalities that lacked clear progression over time. This study, therefore, did not confirm progressive neurodegeneration and regression in the sense of childhood dementia. Long-term studies with larger patient cohorts are required to confirm these results. This study fortified the importance of intensive supportive therapies and adequate technical aids for this patient group. The present data might provide benchmark information to enable critical efficacy assessment of performed HSCT and upcoming novel translational treatments.

## Figures and Tables

**Table 1:** Individual patient characteristics.

Pat. (Sex)	Genotype *GNPTAB* Allele 1/Allele 2	Phenotype	Age at Diagnosis (Years)	Age at First Assessment (Months)	Age at Last Assessment (Months)	No. of Cognitive Tests	No. of Adaptive Behaviour Tests	Age at First MRI (Months)	Age at Last MRI (Months)	No. of MRIs	Age at Death (Years)	Referred to in Ammer et al. [5] as
1 (F)	c.3335 + 1G > A/c.3335 + 1G > A	MLII	0.3	6.6	41.1	7	6	7.3	-	1	N/A	3 (F)
2 (M)	c.3503_3504del/c.3503_3504del	MLII	0.3	9.4	34	4	4	24.8	-	1	N/A	9 (M)
3 (M)	c.3503_3504del/c.3503_3504del	MLII	0.1	1.6	13.7	3	2	0.8	-	1	N/A	7 (M)
4 (F)	c.3503_3504del/c.3503_3504del	MLII	1.9	25.0	26.9	1	1	1.6	-	1	3.1	-
5 (M)	c.3503_3504del/c.1052dup	MLII	2.3	45.6	66.5	3	3	12.1	38.4	3	4.7	4 (M)
6 (F)	c.1001G > A/c.1001G > A	MLII	1.2	27.1	64.1	4	4	10.4	29.3	2	N/A	1 (F)
7 (M)	c.3091C > T/c.344_345del	MLII	0.4	5.6	-	1	1	-	-	-	N/A	-
8 (M)	Unknown	MLII	1.4	12.6	64.7	5	4	39.7	-	1	N/A	14 (M)
9 (F)	c.344_345del/c.1022del	ML intermediate	2.2	135.5	159.6	2	3	16.8	134.7	4	N/A	16 (M)
10 (M)	c.10A > C/c.2502del	ML intermediate	1.3	75.0	-	1	-	-	-	-	11.8	15 (M)
11 (F)	Unknown	MLII	1.0	24.4	-	1	-	-	-	-	3.4	14 (M)

Abbreviations: F, female; M, male; ML, mucolipidosis; MRI, magnet resonance imaging; N/A, not applicable.

**Table 2:** Patient characteristics and developmental outcome.

Characteristic	Overall *N* = 11		MLII *N* = 9		ML Intermediate *N* = 2	
	Mean (SD)	Median (Min–Max)	Mean (SD)	Median (Min–Max)	Mean (SD)	Median (Min–Max)
Patient baseline data						
Body size metrics at birth (*n* = 10)						
Height (cm)	46.1 (6.7)	45.5 (33.5, 58.0)	44.0 (5.4)	45.0 (33.5, 51.0)	54.5 (5.0)	54.5 (51.0, 58.0)
Weight (kg)	2.5 (0.6)	2.3 (1.8, 3.7)	2.3 (0.4)	2.2 (1.8, 3.3)	3.3 (0.7)	3.3 (2.8, 3.7)
OFC (cm)	31.8 (2.9)	32.3 (25.0, 35.0)	31.4 (2.9)	31.9 (25.0, 34.5)	33.8 (1.8)	33.8 (32.5, 35.0)
Body size metrics at last observation (*n* = 10)						
Height (cm)	70.0 (10.0)	68.0 (62.0, 95.0)	66.4 (4.1)	68.0 (62.0, 76.0)	92.5 (3.5)	92.5 (90.0, 95.0)
Weight (kg)	8.4 (3.4)	6.9 (5.9, 18.2)	7.4 (1.7)	6.7 (5.9, 11.5)	15.9 (3.3)	15.9 (13.5, 18.2)
OFC (cm)	45.0 (3.0)	44.5 (42.0, 51.0)	44.1 (2.1)	43.0 (42.0, 48.0)	50.5 (0.7)	50.5 (50.0, 51.0)
Age at diagnosis (months) (*n* = 11)	13.6 (9.7)	14.6 (0.8, 27.7)	11.88 (9.5)	11.73 (0.8, 27.7)	21.3 (7.7)	21.3 (15.9, 26.7)
Developmental assessments						
No. of observations per patient (*n* = 11)	5.5 (3.8)	5.0 (1.0, 13.0)	6.0 (3.9)	6.0 (2.0, 8.0)	3.0 (2.8)	3.0 (1.0, 5.0)
Follow-up period (months)	18.8 (17.7)	20.9 (0.0, 52.1)	20.3 (18.5)	20.9 (0.0, 52.1)	12.1 (17.0)	12.1 (0.0, 24.1)
Overall age (months)	42.72 (37.9)	34.0 (1.6, 159.6)	32.2 (19.2)	29.1 (1.6, 66.5)	137.6 (31.9)	148.1 (75.0, 159.6)
Age at first developmental assessment (months)	30.8 (36.5)	18.5 (1.6, 135.5)	18.3 (15.2)	11.0 (1.6, 45.6)	105.3 (42.8)	105.3 (75.0, 135.5)
Age at last developmental assessment (months)	46.0 (37.9)	37.6 (6.4, 159.6)	35.8 (22.1)	34.0 (6.4, 66.5)	117.3 (59.8)	117.3 (75.0, 159.6)
Neurocognitive assessments only						
No. of observations per patient (*n* = 11)	2.9 (2.0)	3.0 (1.0, 7.0)	3.2 (2.1)	3.0 (1.0, 7.0)	1.5 (0.7)	1.5 (1.0, 2.0)
Follow-up period (months)	17.5 (17.9)	12.1 (0.0, 52.1)	20.1 (18.8)	20.9 (0.0, 52.1)	5.75 (8.1)	5.8 (0.0, 11.5)
At first neurocognitive assessment						
Age (months)	34.9 (43.2)	24.4 (1.6, 148.1)	17.8 (14.2)	12.6 (1.6, 45.6)	111.6 (51.7)	111.6 (75.0, 148.1)
Cognitive ability (Aeqs)	13.3 (18.2)	5.3 (0.5, 59.0)	5.4 (2.6)	5.0 (0.5, 9.0)	48.5 (14.9)	48.5 (38.0, 59.0)
DQ (%)	42.4 (19.4)	39.8 (9.4, 71.9)	41.8 (21.5)	36.9 (9.4, 71.9)	45.3 (7.7)	45.3 (39.8, 50.7)
At last neurocognitive assessment						
Age (months)	52.4 (42.4)	41.1 (6.4, 159.6)	38.0 (22.7)	34.0 (6.4, 66.5)	117.3 (59.8)	117.3 (75.0, 159.6)
Cognitive ability (Aeqs)	18.1 (20.0)	9.0 (4.0, 64.2)	10.8 (11.3)	8.0 (4.0, 40.0)	51.1 (18.5)	51.1 (38.0, 64.2)
DQ (%)	36.7 (20.4)	36.9 (6.2, 71.9)	34.8 (22.1)	29.7 (6.2, 71.9)	45.5 (7.4)	45.5 (40.2, 50.7)
BSID-III only (*n* = 8)						
Age (months)	38.6 (22.5)	37.6 (6.4, 66.5)	38.6 (22.5)	37.6 (6.4, 66.5)	-	-
Recessive language (Aeqs)	9.3 (6.4)	8.0 (3.3, 24.0)	9.3 (6.4)	8.0 (3.3, 24.0)	-	-
Expressive language (Aeqs)	11.2 (6.1)	11.5 (3.6, 20.0)	11.2 (6.1)	11.5 (3.6, 20.0)	-	-
Gross motor skills (Aeqs)	4.4 (2.8)	3.3 (1.3, 10.0)	4.4 (2.8)	3.3 (1.3, 10.0)	-	-
Fine motor skills (Aeqs)	8.6 (4.7)	9.0 (4.3, 17.0)	8.6 (4.7)	9.0 (4.3, 17.0)	-	-
Adaptive behaviour assessments only						
No. of observations per patient (*n* = 9)	3.1 (1.6)	3.0 (1.0, 6.0)	3.1 (1.7)	3.5 (1.0, 6.0)	3.0 (N/A)	3.0 (3.0, 3.0)
Follow-up period (months)	19.0 (13.6)	24.3 (0.0, 35.6)	18.3 (14.4)	21.8 (0.0, 35.6)	24.3 (N/A)	24.3 (24.3, 24.3)
At first adaptive behaviour assessment						
Age (months)	33.8 (40.9)	25.0 (6.4, 135.5)	21.1 (15.8)	17.2 (6.4, 45.6)	135.5 (N/A)	135.5 (135.5, 135.5)
Cognitive ability (Aeqs)	10.5 (12.1)	5.0 (2.0, 41.0)	6.7 (4.3)	5.0 (2.0, 13.9)	41.0 (N/A)	41.0 (41.0, 41.0)
DQ (%)	37.4 (16.6)	30.3 (17.1, 68.5)	38.3 (17.6)	36.3 (17.1, 68.5)	30.3 (N/A)	30.3 (30.3, 30.3)
At last adaptive behaviour assessment						
Age (months)	52.2 (45.5)	41.1 (6.4, 159.1)	38.9 (23.1)	37.6 (6.4, 64.1)	159.1 (N/A)	159.1 (159.1, 159.1)
Cognitive ability (Aeqs)	14.7 (13.2)	10.7 (2.8, 47.3)	10.6 (5.3)	10.5 (2.8, 19.3)	47.3 (N/A)	47.3 (47.3, 47.3)
DQ (%)	34.5 (16.8)	30.6 (8.3, 60.6)	35.1 (17.9)	32.7 (8.3, 60.6)	29.7 (N/A)	29.7 (29.7, 29.7)
Communication (Aeqs)	14.0 (13.6)	12.0 (1.6, 47.0)	9.9 (6.0)	12.0 (1.6, 17.2)	47.0 (N/A)	47.0 (47.0, 47.0)
Activity of daily life (Aeqs)	12.7 (10.4)	9.6 (3.0, 37.2)	9.6 (5.1)	8.5 (3.0, 19.6)	37.2 (N/A)	37.2 (37.2, 37.2)
Social skills (Aeqs)	18.9 (16.1)	15.5 (3.5, 59.0)	13.9 (8.1)	14.4 (3.5, 22.2)	59.0 (N/A)	59.0 (59.0, 59.0)
Motor skills (Aeqs)	6.4 (3.9)	5.8 (2.0, 13.5)	6.4 (3.9)	5.8 (2.0, 13.5)	-	-

Abbreviations: Aeqs, age-equivalent scores; BSID-III, Bayley Scales of Infant and Toddler Development-III; DQ, developmental quotient; max, maximum; min, minimum; ML, mucolipidosis; N/A, not applicable; OFC, occipitofrontal circumference.

**Table 3:** Neuroimaging findings in patients with MLII and ML intermediate.

Characteristic	Overall *N* = 8 *n* (%)	MLII *N* = 7 *n* (%)	ML Intermediate *N* = 1 *n* (%)
Cervical spinal canal			
Mild stenosis, 0.55–0.80 cm	3 (38)	3 (43)	-
Moderate stenosis, 0.50 cm	1 (13)	1 (14)	-
Condition after cervical spine decompression	1 (13)	-	1 (100)
White matter			
Myelinization mildly lagging behind	2 (25)	2 (29)	-
Atrophy of the white matter	4 (50)	4 (57)	-
White matter lesions, rather progressive	3 (38)	2 (29)	1 (100)
Brain volume and cerebrospinal fluid spaces			
Mild cerebral atrophy, milder over time	3 (38)	3 (43)	-
Mild atrophy of the cerebellum	1 (13)	-	1 (100)
Mildly enlarged subarachnoid spaces, non-progressive	3 (38)	2 (29)	1 (100)
Plump ventricles I-III	2 (25)	2 (29)	-
Asymmetry of the lateral ventricles	2 (25)	1 (14)	1 (100)
Mildly enlarged Virchow-Robin’s spaces, non-progressive	2 (25)	1 (14)	1 (100)
Sporadic abnormalities			
Pineal gland cyst	2 (25)	1 (14)	1 (100)
Cerebellar tonsil depression	1 (13)	1 (14)	-
Caput quadratum with a seemingly small frontal brain	1 (13)	1 (14)	-
Dysplastic corpus callosum, slender commissura antrior, prominent adhaesio interthalamica	1 (13)	1 (14)	-

## Data Availability

The data that support the findings of this study are available from the corresponding author upon reasonable request. The data are not publicly available, to ensure that the privacy of the study patients with rare diseases is not compromised.

## Supplementary Materials

The following supporting information can be downloaded at: https://www.mdpi.com/article/10.3390/jcm12124114/s1, Figure S1: Correlation of age-equivalent scores of Vineland Adaptive Behaviour Scales-II and Bayley Scales of Infant and Toddler Development-III. Table S1: Raw data from developmental assessments of MLII and ML intermediate patients.

## References

[1] Tiede S., Storch S., Lübke T., Henrissat B., Bargal R., Raas-Rothschild A., Braulke T. (2005). Mucolipidosis II is caused by mutations in *GNPTA* encoding the α/β GlcNAc-1-phosphotransferase. Nat. Med..

[2] Kollmann K., Pohl S., Marschner K., Encarnação M., Sakwa I., Tiede S., Poorthuis B.J., Lübke T., Müller-Loennies S., Storch S. (2010). Mannose phosphorylation in health and disease. Eur. J. Cell Biol..

[3] Velho R.V., Harms F.L., Danyukova T., Ludwig N.F., Friez M.J., Cathey S.S., Filocamo M., Tappino B., Güneş N., Tüysüz B. (2019). The lysosomal storage disorders mucolipidosis type II, type III alpha/beta, and type III gamma: Update on *GNPTAB* and *GNPTG* mutations. Hum. Mutat..

[4] Dogterom E.J., Wagenmakers M., Wilke M., Demirdas S., Muschol N.M., Pohl S., van der Meijden J.C., Rizopoulos D., van der Ploeg A.T., Oussoren E. (2021). Mucolipidosis type II and type III: A systematic review of 843 published cases. Anesth. Analg..

[5] Ammer L.S., Oussoren E., Muschol N.M., Pohl S., Rubio-Gozalbo M.E., Santer R., Stuecker R., Vettorazzi E., Breyer S.R. (2020). Hip Morphology in Mucolipidosis Type II. J. Clin. Med..

[6] David-Vizcarra G., Briody J., Ault J., Fietz M., Fletcher J., Savarirayan R., Wilson M., McGill J., Edwards M., Munns C. (2010). The natural history and osteodystrophy of mucolipidosis types II and III. J. Paediatr. Child Health.

[7] Ammer L.S., Muschol N.M., Santer R., Lang A., Breyer S.R., Sasu P.B., Petzoldt M., Dohrmann T. (2022). Anaesthesia-Relevant Disease Manifestations and Perianaesthetic Complications in Patients with Mucolipidosis—A Retrospective Analysis of 44 Anaesthetic Cases in 12 Patients. J. Clin. Med..

[8] Di Lorenzo G., Westermann L.M., Yorgan T.A., Stürznickel J., Ludwig N.F., Ammer L.S., Baranowsky A., Ahmadi S., Pourbarkhordariesfandabadi E., Breyer S.R. (2021). Pathogenic variants in *GNPTAB* and *GNPTG* encoding distinct subunits of GlcNAc-1-phosphotransferase differentially impact bone resorption in patients with mucolipidosis type II and III. Genet. Med. Off. J. Am. Coll. Med. Genet..

[9] Cathey S.S., Leroy J.G., Wood T., Eaves K., Simensen R.J., Kudo M., Stevenson R.E., Friez M.J. (2010). Phenotype and genotype in mucolipidoses II and III alpha/beta: A study of 61 probands. J. Med. Genet..

[10] Shibazaki T., Hirabayashi K., Saito S., Shigemura T., Nakazawa Y., Sakashita K., Takagi M., Shiohara M., Adachi K., Nanba E. (2016). Clinical and laboratory outcomes after umbilical cord blood transplantation in a patient with mucolipidosis II alpha/beta. Am. J. Med. Genet. Part A.

[11] Kollmann K., Damme M., Markmann S., Morelle W., Schweizer M., Hermans-Borgmeyer I., Röchert A.K., Pohl S., Lübke T., Michalski J.-C. (2012). Lysosomal dysfunction causes neurodegeneration in mucolipidosis II ‘knock-in’ mice. Brain.

[12] Idol R.A., Wozniak D.F., Fujiwara H., Yuede C.M., Ory D.S., Kornfeld S., Vogel P. (2014). Neurologic abnormalities in mouse models of the lysosomal storage disorders mucolipidosis II and mucolipidosis III γ. PLoS ONE.

[13] Favret J.M., Weinstock N.I., Feltri M.L., Shin D. (2020). Pre-clinical Mouse Models of Neurodegenerative Lysosomal Storage Diseases. Front. Mol. Biosci..

[14] Hoogerbrugge P.M., Brouwer O.F., Bordigoni P., Ringden O., Kapaun P., Ortega J.J., O’Meara A., Cornu G., Souillet G., Frappaz D. (1995). Allogeneic bone marrow transplantation for lysosomal storage diseases. Lancet.

[15] De Ru M.H., Boelens J.J., Das A.M., Jones S.A., van der Lee J.H., Mahlaoui N., Mengel E., Offringa M., O’Meara A., Parini R. (2011). Enzyme replacement therapy and/or hematopoietic stem cell transplantation at diagnosis in patients with mucopolysaccharidosis type I: Results of a European consensus procedure. Orphanet J. Rare Dis..

[16] Köhn A.F., Grigull L., du Moulin M., Kabisch S., Ammer L., Rudolph C., Muschol N.M. (2020). Hematopoietic stem cell transplantation in mucopolysaccharidosis type IIIA: A case description and comparison with a genotype-matched control group. Mol. Genet. Metab. Rep..

[17] Ammer L.S., Pohl S., Breyer S.R., Aries C., Denecke J., Perez A., Petzoldt M., Schrum J., Müller I., Muschol N.M. (2021). Is hematopoietic stem cell transplantation a therapeutic option for mucolipidosis type II?. Mol. Genet. Metab. Rep..

[18] Lund T.C., Cathey S.S., Miller W.P., Eapen M., Andreansky M., Dvorak C.C., Davis J.H., Dalal J.D., Devine S.M., Eames G.M. (2014). Outcomes after hematopoietic stem cell transplantation for children with I-cell disease. Biol. Blood Marrow Transplant..

[19] Ko A.-R., Jin D.-K., Cho S.Y., Park S.W., Przybylska M., Yew N.S., Cheng S.H., Kim J.-S., Kwak M.J., Kim S.J. (2016). AAV8-mediated expression of N-acetylglucosamine-1-phosphate transferase attenuates bone loss in a mouse model of mucolipidosis II. Mol. Genet. Metab..

[20] Matos L., Vilela R., Rocha M., Santos J.I., Coutinho M.F., Gaspar P., Prata M.J., Alves S. (2020). Development of an Antisense Oligonucleotide-Mediated Exon Skipping Therapeutic Strategy for Mucolipidosis II: Validation at RNA Level. Hum. Gene Ther..

[21] Westermann L.M., Baranowsky A., Di Lorenzo G., Danyukova T., Soul J., Schwartz J.-M., Hendrickx G., Amling M., Rose-John S., Garbers C. (2021). Transgenic inhibition of interleukin-6 trans-signaling does not prevent skeletal pathologies in mucolipidosis type II mice. Sci. Rep..

[22] Shapiro E.G., Escolar M.L., Delaney K.A., Mitchell J.J. (2017). Assessments of neurocognitive and behavioral function in the mucopolysaccharidoses. Mol. Genet. Metab..

[23] Bates D., Mächler M., Bolker B., Walker S. (2015). Fitting Linear Mixed-Effects Models Using lme4. J. Stat. Softw..

[24] Leroy J.G., Sillence D., Wood T., Barnes J., Lebel R.R., Friez M.J., Stevenson R.E., Steet R., Cathey S.S. (2013). A novel intermediate mucolipidosis II/IIIαβ caused by *GNPTAB* mutation in the cytosolic N-terminal domain. Eur. J. Hum. Genet..

[25] Shapiro E.G., Nestrasil I., Delaney K.A., Rudser K., Kovac V., Nair N., Richard C.W., Haslett P., Whitley C.B. (2016). A Prospective Natural History Study of Mucopolysaccharidosis Type IIIA. J. Pediatr..

[26] Shapiro E.G., Whitley C.B., Eisengart J.B. (2018). Beneath the floor: Re-analysis of neurodevelopmental outcomes in untreated Hurler syndrome. Orphanet J. Rare Dis..

[27] Tabone L., Caillaud C., Amaddeo A., Khirani S., Michot C., Couloigner V., Brassier A., Cormier-Daire V., Baujat G., Fauroux B. (2019). Sleep-disordered breathing in children with mucolipidosis. Am. J. Med. Genet. Part A.

[28] Leroy J.G., Spranger J.W., Feingold M., Opitz J.M., Crocker A.C. (1971). I-cell disease: A clinical picture. J. Pediatr..

[29] Libert J., Van Hoof F., Farriaux J.-P., Toussaint D. (1977). Ocular findings in I-cell disease (mucolipidosis type II). Am. J. Ophthalmol..

[30] Gottlieb D.J., Chase C., Vezina R.M., Heeren T.C., Corwin M.J., Auerbach S.H., Weese-Mayer D.E., Lesko S.M. (2004). Sleep-disordered breathing symptoms are associated with poorer cognitive function in 5-year-old children. J. Pediatr..

[31] Marcus C.L., Radcliffe J., Konstantinopoulou S., Beck S.E., Cornaglia M.A., Traylor J., DiFeo N., Karamessinis L.R., Gallagher P.R., Meltzer L.J. (2012). Effects of positive airway pressure therapy on neurobehavioral outcomes in children with obstructive sleep apnea. Am. J. Respir. Crit. Care Med..

[32] Scott-Warren V.L., Walker R. (2020). Perioperative management of patients with Mucolipidosis II and III: Lessons from a case series. Pediatr. Anesth..

[33] Nicolas-Jilwan M., AlSayed M. (2018). Mucopolysaccharidoses: Overview of neuroimaging manifestations. Pediatr. Radiol..

[34] Edmiston R., Wilkinson S., Jones S., Tylee K., Broomfield A., Bruce I.A., Morava E., Baumgartner M., Patterson M., Rahman S., Zschocke J., Peters V. (2019). I-Cell Disease (Mucolipidosis II): A Case Series from a Tertiary Paediatric Centre Reviewing the Airway and Respiratory Consequences of the Disease. JIMD Reports.

[35] Alegra T., Sperb-Ludwig F., Guarany N.R., Ribeiro E.M., Lourenço C.M., Kim C.A., Valadares E.R., Galera M.F., Acosta A.X., Horovitz D.D.G. (2019). Clinical Characterization of Mucolipidoses II and III: A Multicenter Study. J. Pediatr. Genet..

[36] Martin J.J., Leroy J.G., Van Eygen M., Ceuterick C. (1984). I-cell disease. A further report on its pathology. Acta Neuropathol..

[37] Bosshard N.U., Hubler M., Arnold S., Briner J., Spycher M.A., Sommerlade H.-J., von Figura K., Gitzelmann R. (1996). Spontaneous mucolipidosis in a cat: An animal model of human I-cell disease. Veter.-Pathol..

