# Supplementary material for: CNS Manifestations in Mucolipidosis Type II—A Retrospective Analysis of Longitudinal Data on Neurocognitive Development and Neuroimaging in Eleven Patients
Source: J Clin Med. 2023 Jun 18;12(12):4114. doi: 10.3390/jcm12124114 (PMC10299287; doi:10.3390/jcm12124114)
Supplement: Supplementary file 1 [file jcm-12-04114-s001.zip › jcm-2431947-supplementary.pdf]

Journal of
Clinical Medicine

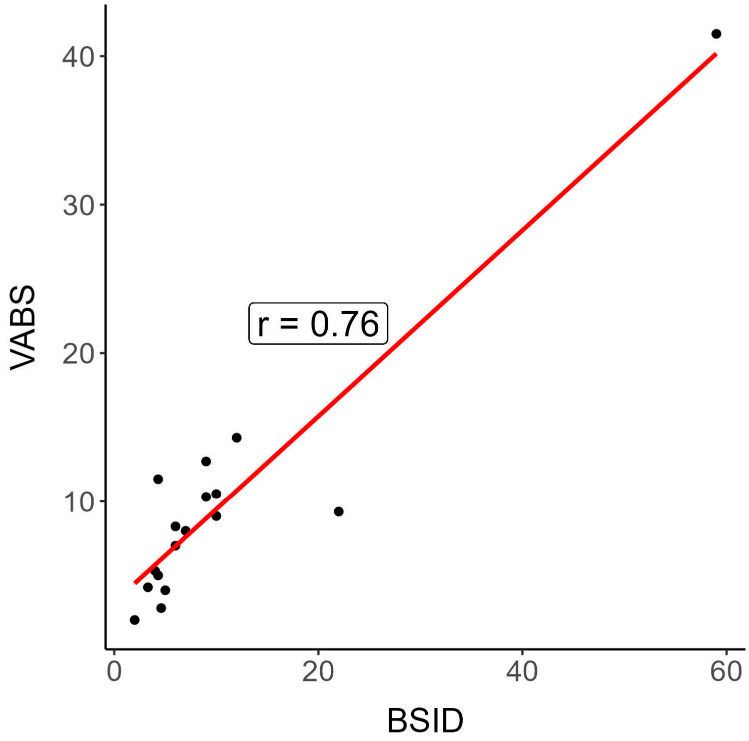

Figure S1. Correlation of age-equivalent scores of Vineland Adaptive Behavior Scales-II (VABS) and Bayley Scales of Infant and Toddler Development-III (BSID). Only age equivalent results of MLII patients (n = 7), who had both assessments performed on one day, were included. "r" is the Spearman correlation coefficient.

Table S1. Raw data from developmental assessments of MLII and ML intermediate patients.

Pat.	Test	Age	Cognitive		DQ	Recessive language		Expressive language		Fine motor		Gross motor		Verbal		Daily		Social		Motor	
		Mon.	Raw	Aeqs	(%)	Raw	Aeqs	Raw	Aeqs	Raw	Aeqs	Raw	Aeqs	SS	Aeqs	SS	Aeqs	SS	Aeqs	SS	Aeqs
1	Griffith	6.6		4	60.6																
1	BSID-III	7.3	21	4.3	58.9	7	3.3	6	3.6	16	4.6	10	2.3								
1	VABS-II	7.3		5	68.5									94	6	74	4	92	6	62	3
1	BSID-III	12.5	29	6	48.0	8	4.3	10	9	22	7	21	5.3								
1	VABS-II	12.5		7	56.0									80	7	62	7	74	7	60	5
1	BSID-III	19.9	39	10	50.3	10	8	12	10	25	9	19	5								
1	VABS-II	19.9		9	45.2									64	9	70	10	71	9	50	6.5
1	BSID-III	28.8	36	9	31.3	11	8	14	12	29	13	20	5.3								
1	VABS-II	28.8		12.7	44.1									65	11.5	73	14.3	76	12.3	44	7.5
1	VABS-II	33.9		16.1	47.5									53	12	74	17.3	84	19	50	10.5
1	BSID-III	36.6	39	10	27.3	13	10	15	13	28	12	15	3.7								
1	VABS-II	41.1		14.3	34.8									60	12	56	13.3	77	17.6	41	10
1	BSID-III	41.1	42	12	29.2	13	10	17	15												
2	BSID-III	9.4	26	5	53.2	8	4.3	7	5.3	19	5.3	10	2.3								
2	VABS-II	9.4		4	42.6									72	5	64	4	62	3	56	3.5
2	BSID-III	20.7	30	7	33.8	9	5.3	8	7	23	8	16	3.6								
2	VABS-II	20.7		8	38.6									62	9	54	8	70	8	38	4.5
2	BSID-III	28.1	38	10	35.6	12	9	15	13	24	9	23	6								
2	VABS-II	28.1		10.5	37.4									57	8.6	62	15.6	84	8	47	8
2	BSID-III	34.0	35	9	26.5	12	9	14	12	26	10	22	6								
2	VABS-II	34.0		10.3	30.3									48	15.5	56	7.3	74	13.3	38	7
3	BSID-III	1.6	4	0.5	31.3	3	0.5	3	1	2	0.5	4	0.5								
3	BSID-III	6.7	9	2	29.9	9	5.3	9	8	9	3.3	10	2.3								

3	VABS-II	6.7		2	29.9									91	4	54	1	72	1	64	3
3	VABS-II	13.7		8.3	60.6									64	5.5	56	7.3	95	12	50	4.5
3	BSID-III	13.7	29	6	43.8	11	8	13	11	24	9	15	3.7								
4	VABS-II	25.0		13.9	55.6									75	13	66	9.6	93	19	47	7.5
4	BSID-III	26.9	32	8	29.7	10	8	8	7	27	11	14	3.3								
5	BSID-III	45.6	22	4.3	9.4	11	8	14	12	17	5	11	2.6								
5	VABS-II	45.6		11.5	25.2									52	9.5	38	7.6	75	17.3	20	3
5	VABS-II	56.2		13	23.1									38	11	36	12	60	15	20	5
5	BSID-III	57.8	25	5	8.7	10	8	13	11	19	5.3	10	2.3								
5	VABS-II	63.6		10.7	16.8									30	12	30	10.6	44	15.5	20	3
5	BSID-III	66.5	23	4.6	6.9	11	8	21	17	13	4.3	7	1.3								
6	BSID-III	27.1	28	5.3	19.6	8	4.3	7	5.3	22	7	8	1.6								0
6	VABS-II	29.3		5	17.1									23	2.5	58	7	52	5	29	3
6	BSID-III	44.1	15	3.3	7.5	6	2	3	1	11	3.6	10	2.3								
6	VABS-II	41.1		4.5	10.9									20	1.5	42	7	40	5	20	3
6	BSID-III	51.2	15	3.3	6.4	6	2	8	7	12	4	14	3.3								
6	VABS-II	51.2		4.2	8.2									20	1	36	6.3	36	5.3	20	4
6	VABS-II	64.1		5.3	8.3									20	1.6	26	6.1	40	8.1	20	4
6	BSID-III	64.1	19	4	6.2	7	3.3	6	3.7	16	4.7	13	3.3								
7	BSID-III	6.4	23	4.6	71.9	8	4.3	6	3.6	12	4.3	12	3								
7	VABS-II	6.4		2.8	43.8									75	2	74	3	77	3.5	50	2
8	BSID-II	12.6		8	63.5																
8	BSID-III	38.9	62	22	56.6	17	15	21	17	36	19	33	9								
8	VABS-II	38.9		9.3	23.9									58	10	52	11	52	7	47	10
8	VABS-II	44.0		17.3	39.3									54	13	68	24	68	15	41	10.5
8	BSID-III	49.0	64	23	46.9																
8	VABS-II	51.0		19.3	37.8									63	19.5	64	23	66	15.5	47	14.5
8	BSID-III	55.9	72	29	51.9	28	24	24	20	34	17	35	10								

8	VABS-II	63.0		19.3	30.6									42	17.2	48	19.6	68	22.2	35	13.5
8	SON 2-7	64.7		40	61.8																
9	VABS-II	135.5		41	30.3									42	40	26	29	68	55		
9	KABC-II	148.1		59	39.8																
9	VABS-II	148.1		41.5	41.5									42	44	24	34	56	46.6		
9	VABS-II	159.1		47.3	29.7									44	47	24	37.2	62	59		
9	KABC-II	159.6		64.2	40.2																
10	SON 2-7	75.0		38	50.7																
11	Griffith	24.4		9	36.9																

Abbreviations: Aeqs, age- equivalent score; BSID, Bayley Scales of Infant and Toddler Development-II/III; DQ, developmental quotient; GMDS, Griffith Scales of Child Development-III; KABC, Kaufman Assessment Battery for Children-II; Raw, raw score; SON, Snijders-Oomen Nonverbal Intelligence Test-2.5-7; SS, standard score; VABS, Vineland Adaptive Behavior Scales-II.
